# Parameter estimation and prediction for coronavirus disease outbreak 2019 (COVID-19) in Algeria

**DOI:** 10.3934/publichealth.2020026

**Published:** 2020-05-22

**Authors:** Soufiane Bentout, Abdennasser Chekroun, Toshikazu Kuniya

**Affiliations:** 1 Department of Mathematics and Informatics, University center of Ain Temouchent, Algeria; 2 Laboratoire d'Analyse Nonlinéaire et Mathématiques Appliquées, University of Tlemcen, Tlemcen 13000, Algeria; 3 Graduate School of System Informatics, Kobe University, 1-1 Rokkodai-cho, Nada-ku, Kobe 657-8501, Japan

**Keywords:** COVID-19, SEIR epidemic model, basic reproduction number

## Abstract

**Background:**

The wave of the coronavirus disease outbreak in 2019 (COVID-19) has spread all over the world. In Algeria, the first case of COVID-19 was reported on 25 February, 2020, and the number of confirmed cases of it has increased day after day. To overcome this difficult period and a catastrophic scenario, a model-based prediction of the possible epidemic peak and size of COVID-19 in Algeria is required.

**Methods:**

We are concerned with a classical epidemic model of susceptible, exposed, infected and removed (SEIR) population dynamics. By using the method of least squares and the best fit curve that minimizes the sum of squared residuals, we estimate the epidemic parameter and the basic reproduction number *ℜ*_0_. Moreover, we discuss the effect of intervention in a certain period by numerical simulation.

**Results:**

We find that *ℜ*_0_ = 4.1, which implies that the epidemic in Algeria could occur in a strong way. Moreover, we obtain the following epidemiological insights: the intervention has a positive effect on the time delay of the epidemic peak; the epidemic size is almost the same for a short intervention; a large epidemic can occur even if the intervention is long and sufficiently effective.

**Conclusion:**

Algeria must implement the strict measures as shown in this study, which could be similar to the one that China has finally adopted.

## 1 Introduction

The mathematical models in epidemiology have been used to understand the temporal dynamics of infectious diseases. The first model used to study the spread of infectious diseases was given by Kermack and Mckendrick [1] in 1927. Practically, this model is based on a system of ordinary differential equations and has been widely investigated with several modifications, in [2]–[7] and references therein. The distinct variables to formulate the individuals compartments are susceptible (S), exposed (E), infected (I) and recovered (or removed, R). The classical SEIR model has been widely studied, for instance, see [8]–[13]. It is shown that the asymptotic behavior depends on the basic reproduction number *ℜ*_0_ (the expected number of secondary cases produced by an infective person in a completely susceptible population). It is described as a threshold value that indicates whether or not the initial outbreak occurs. That is, if *ℜ*_0_ < 1, then the infective population tends to decrease and there is no outbreak, whereas if *ℜ*_0_ > 1, then the infective population tends to increase and an outbreak occurs.

In December 2019, the first case of a novel coronavirus disease was recognized at Wuhan in China [14]. The wave of this disease has spread all over the world, and the World Health Organization (WHO) named it the coronavirus disease outbreak in 2019 (COVID-19) on 11 February, 2020 [14]. In China, during the period from December 2019 to 31 January, 2020, about 10 thausand (9,720) cases of COVID-19 were confirmed [14]. We have to note that asymptomatic individuals of COVID-19 can transmit the infection [15]. Therefore, there would be more cases that could not be reported by medical authorities. In the absence of effective vaccines and therapeutics against COVID-19, countries have to resort to non-pharmaceutical interventions to avoid the infection or to slow down the spread of the epidemic.

In Algeria, the first case was reported on 25 February 2020 [16]. Since then, the number of confirmed cases of COVID-19 has increased day after day. From the end of March, 2020, the Algerian government mandated several approaches to eradicate the spread of COVID-19 such as trying to control the source of contagion and reducing the number of contacts between individuals by confinement and isolation [17]. The purpose of this study is to know how the epidemic will evolve in Algeria with and without such interventions. We use the early data reported in [18] until 31 March, 2020. Recently, many works used a mathematical models for COVID-19, for instance, see the following contributions [19]–[23]. In [20], an SEIR epidemic model with partially identified infected individuals was used for the prediction of the epidemic peak of COVID-19 in Japan. The results in [20] were restricted only to the cases in Japan, and the applicability of the model to the cases in any other countries were not discussed. In this paper, we apply a similar SEIR epidemic model as in [20] to the cases in Algeria. This work would contribute not only in understanding the possible spread pattern of COVID-19 in Algeria in order to act appropriately to reduce the epidemic damage, but also in showing the applicability of the model-based approach as in [20] to the cases in other countries, which might help us to assess the epidemic risk of COVID-19 worldwide in future.

## 2 Materials and method

### 2.1 Data

**Figure 1.**
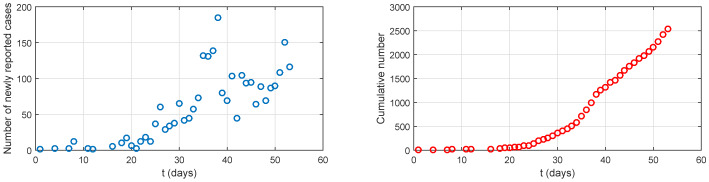
Daily reported number of new cases (left) and accumulated cases (right) of COVID-19 in Algeria from 25 February to 18 April, 2020 [18].

We use the data of confirmed COVID-19 cases in Algeria, which is available in the epidemiological map in [18]. The data consists of the daily reported number of new cases and accumulated cases for COVID-19 in Algeria from 25 February to 18 April, 2020 (see Figure 1 and Table 1). The number of reported cases has increased rapidly in the exponential sense until the beginning of April.

**Table 1.** Number of newly reported cases and cumulative number of COVID-19 in Algeria from 25 February to 18 April, 2020 with the nationwide isolation. From 25 February to 18 April 2020.

Date (day/month)	Number of newly reported cases	Cumulative number
25 February	1	1
26 February	0	1
27 February	0	1
28 February	0	1
29 February	2	3
1 March	0	3
2 March	0	3
3 March	2	5
4 March	12	17
5 March	0	17
6 March	0	17
7 March	2	19
8 March	1	20
9 March	0	20
10 March	0	20
11 March	0	20
12 March	5	25
13 March	0	25
14 March	10	35
15 March	17	52
16 March	6	58
17 March	2	60
18 March	12	72
19 March	18	90
20 March	12	102
21 March	37	139
22 March	60	201
23 March	29	230
24 March	34	264
25 March	38	302
26 March	65	367
27 March	42	409
28 March	45	454
29 March	57	511
30 March	73	584
31 March	132	716
1 April	131	847
2 April	139	986
3 April	185	1171
4 April	80	1251
5 April	69	1320
6 April	103	1423
7 April	45	1468
8 April	104	1572
9 April	94	1666
10 April	95	1761
11 April	64	1825
12 April	89	1914
13 April	69	1983
14 April	87	2070
15 April	90	2160
16 April	108	2268
17 April	150	2418
18 April	116	2534

### 2.2 Model

**Figure 2.**
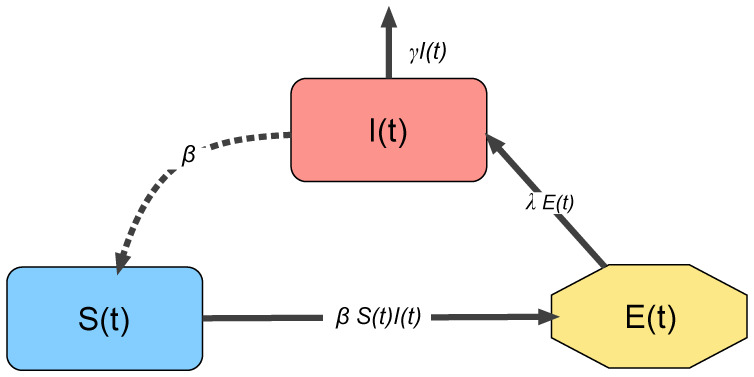
Interactions between the compartments of the epidemiological model (1). The continuous lines represent transition between compartments, and entrance and exit of individuals. The dashed line represents the transmission of the infection through the interaction between susceptible and infected individuals. The recovered class is omitted because it is decoupled from the other compartments.

In this paper, we use the following well-known SEIR epidemic model, for *t* > 0,

$$\{\begin{array}{c} S^{\prime }\left(t\right)=-\beta S\left(t\right)I\left(t\right),\\ E^{\prime }\left(t\right)=\beta S\left(t\right)I\left(t\right)-\lambda E\left(t\right),\\ I^{\prime }\left(t\right)=-\gamma I\left(t\right)+\lambda E\left(t\right),\\ R^{\prime }\left(t\right)=\gamma I\left(t\right), \end{array}$$ (1)

with initial conditions

$$S\left(0\right)=S_{0},\;E\left(0\right)=E_{0},\;I\left(0\right)=I_{0}\text{ and }R\left(0\right)=R_{0}.$$ (2)

(1)–(2) is a system of ordinary differential equations based on the phenomenological law of mass action. For simplicity, we suppose that *E*_0_ = *R*_0_ = 0 (initially, there is no exposed and recovered individual). Moreover, we assume that *S*(0) + *E*(0) + *I*(0) + *R*(0) = 1 from which we have *S*(*t*) + *E*(*t*) + *I*(*t*) + *R*(*t*) = 1 for all *t* > 0, and hence, each population implies the proportion to the total population. All the parameters of the model are nonnegative constants, and they are described in Table 2. Figure 2 provides a schematic representation of model (1).

Note that *γ* implies the removal rate and the removed population *R* includes the individuals who died due to the infection.

We follow the same idea in [20] to give the prediction for Algeria. Parameter estimation and epidemic peak are treated and obtained. Moreover, we estimate the basic reproduction number for the epidemic COVID-19 in Algeria. Since the virus presents asymptomatic cases and the fact that there is a sufficiently lack of diagnostic test, we consider an identification function

$$t\in {ℜ}^{+}\mapsto X\left(t\right)=\varepsilon \times I\left(t\right)\times N.$$ (3)

This quantity describes the number of infective individuals who are identified at time *t*, with *N* is the total population in Algeria (*N* = 43411571) and *ε* is the identification rate. As in [20], we suppose that *ε* ∈ [0.01, 0.1].

## 3 Results

### 3.1 Epidemic prediction

In this section, we develop simulations to provide epidemic predictions for the COVID-19 epidemic in Algeria. We focus on predicting the cases and parameter estimation. We are able to find the basic reproduction number and to estimate the infection rate. Recall that the number *ℜ*_0_ is defined as the average number of secondary infections that occur when one infective individual is introduced into a completely susceptible population. In epidemiology, the method to compute the basic reproduction number using the next-generation matrix is given by Diekmann et al. [24] and Van den Driessche and Watmough [25]. For our model, the value of the basic reproduction number of the disease is defined by

$${ℜ}_{0}=\frac{\beta S_{0}}{\gamma }=\frac{\beta }{\gamma }\left(1-E_{0}-I_{0}-R_{0}\right)=\frac{\beta }{\gamma }(1-\frac{X\left(0\right)}{\varepsilon N}).$$ (4)

In fact, the largest eigenvalue or spectral radius of *FV*^−1^ is the basic reproduction number of the model, where

$$F=\left[\begin{array}{cc} 0 & \beta S_{0}\\ 0 & 0 \end{array}\right]\text{ and }V=\left[\begin{array}{cc} \lambda & 0\\ -\lambda & \gamma \end{array}\right].$$ (5)

In our case, we assume that *X*(0) = 1 and *N* = 43411571, see Tables 1 and 2.

**Figure 3.**
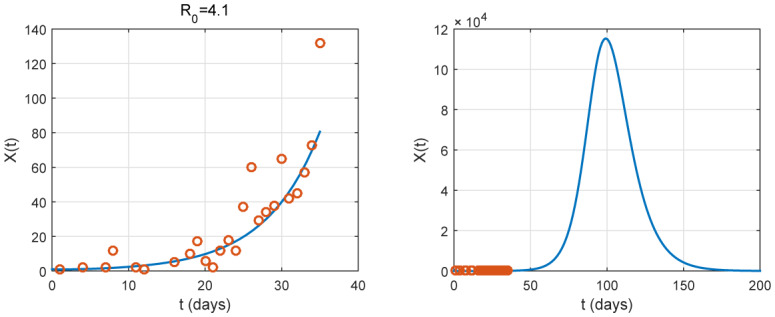
Graph of *X*(*t*), with *ε* = 0.01, is plotted. It represents the number of identified newly cases. The red small circles are the reported case data. In this case, the data from the number of newly reported cases is well fitted the epidemic. Without the nationwide lockdown, the peak occurs approximately at *t* = 100 associated to a date between the beginning and the middle of June.

**Figure 4.**
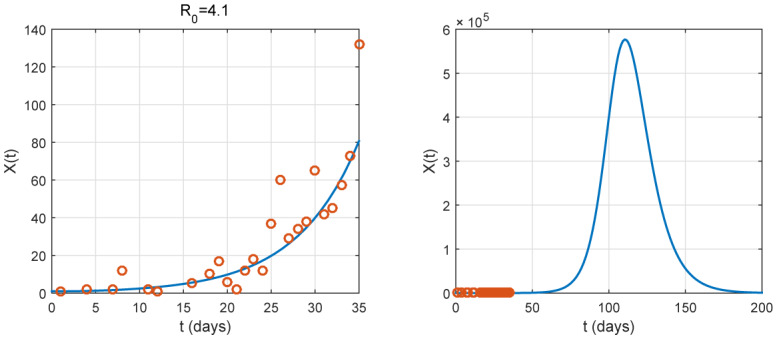
Graph of *X*(*t*), with *ε* = 0.05, is plotted. It represents the number of identified newly cases. The red small circles are the reported case data. In this case, the data from the number of newly reported cases is well fitted the epidemic. Without the nationwide lockdown, the peak occurs approximately at *t* = 110 associated to a date close to the middle of June.

**Figure 5.**
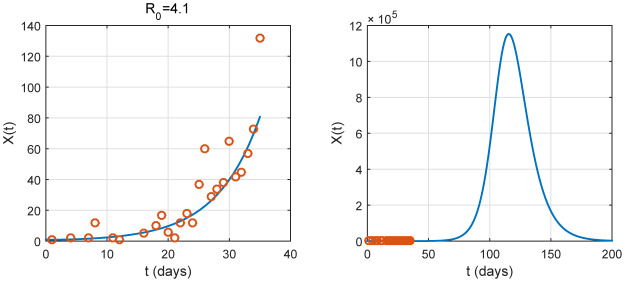
Graph of *X*(*t*), with *ε* = 0.1, is plotted. It represents the number of identified newly cases. The red small circles are the reported case data. In this case, the data from the number of newly reported cases is well fitted the epidemic. Without the nationwide lockdown, the peak occurs approximately at *t* = 115 associated to a date between the middle and the end of June.

By some choices on the parameter *ε*, illustrations for prediction are given in Figures 3, 4 and 5. To estimate the parameters, we use the method of least squares and the best fit curve that minimizes the sum of squared residuals. We remark that *ε* does not affect the basic reproduction number and the infection rate as the total population *N* is large. We obtain an estimation of them as shown in Table 2 (*ℜ*_0_ = 4.1 and *β* = 0.41). However, we observe that *ε* is an important parameter for prediction. In fact, the three illustrations in Figures 3, 4 and 5 show its influence on the peak.

As stated before, we use a simple but useful measure to provide the average number of infections caused by one infected individual *R*_0_ = 4.1. The *R*_0_ value in China was estimated to be around 2.5 in the early stage of epidemic. In April 2020, the contagiousness rate was reassessed upwards, between 3.8 and 8.9 (see, [26]). Comparing with other results (see, [27]), *R*_0_ may be unstable.

**Table 2.** Parameter values for numerical simulation.

Description	Value	Reference
*β*: Contact rate	0.41	Estimated
*γ*: Removal rate	0.1	[28], [29]
*λ*: Onset rate	0.2	[14], Situation report 30, [28], [30]
1/*γ*: The average infectious period	10	[28], [29]
1/*λ*: The average incubation period	5	[14], Situation report 30, [28], [30]
*ε*: Identification rate	0.01–0.1	[20]
*N*: Total population in Algeria	43411571	[31]
*ℜ*_0_: Basic reproduction number	4.1	Estimated

### 3.2 Intervention effect

**Figure 6.**
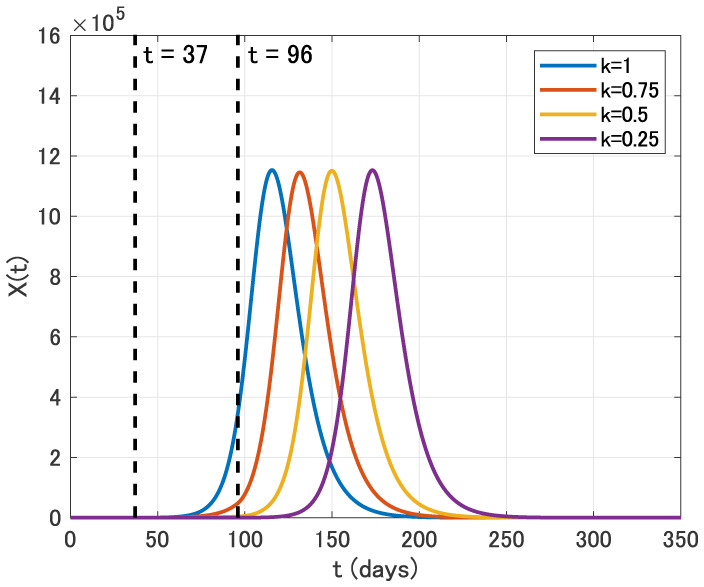
Time variation of the identification function *X*(*t*) for *k* = 1, 0.75, 0.5 and 0.25 in the two months intervention from April 1 (*t* = 37) to May 31 (*t* = 96).

We now discuss the effect of intervention. We first assume that the intervention is carried out for two months from April 1 (*t* = 37) to May 31 (*t* = 96) with reducing the contact rate *β* to *kβ*, where 0 ≤ *k* ≤ 1. We use the parameter values as in Table 2 with *ε* = 0.1. Time variation of the identification function *X*(*t*) for *k* = 1, 0.75, 0.5 and 0.25 is displayed in Figure 6.

From Figure 6, we see that the two months intervention in this case has the positive effect on the time delay of the epidemic peak. On the other hand, the epidemic size is almost the same for each *k* in this case.

We secondly assume that the intervention is carried out for three months from April 1 (*t* = 37) to June 30 (*t* = 126).

**Figure 7.**
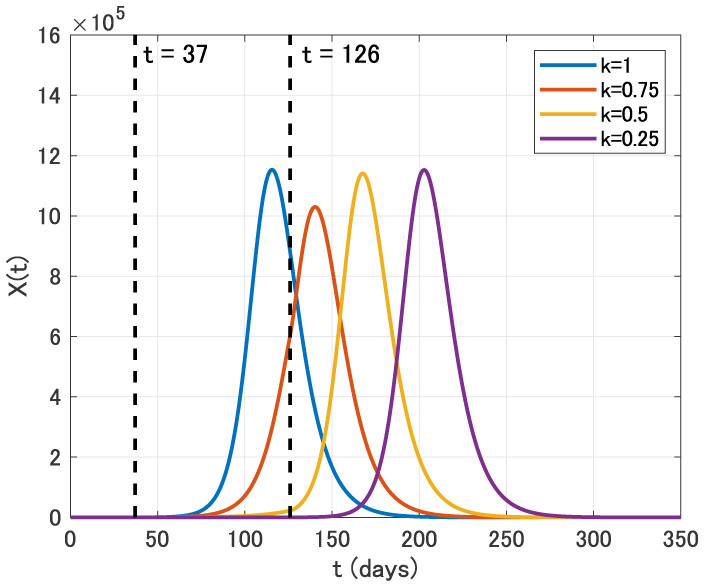
Time variation of the identification function *X*(*t*) for *k* = 1, 0.75, 0.5 and 0.25 in the three months intervention from April 1 (*t* = 37) to June 30 (*t* = 126).

From Figure 7, we see that the epidemic peak is also delayed in this case. Moreover, the epidemic size is reduced for *k* = 0.75. In contrast, the epidemic size for *k* = 0.5 and *k* = 0.25 is almost the same as that for *k* = 1.

We thirdly assume that the intervention is carried out for four months from April 1 (*t* = 37) to July 31 (*t* = 157).

**Figure 8.**
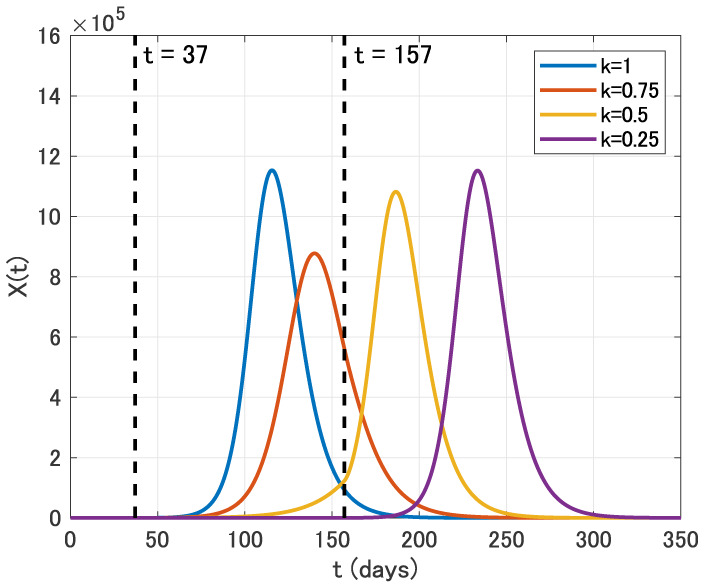
Time variation of the identification function *X*(*t*) for *k* = 1, 0.75, 0.5 and 0.25 in the four months intervention from April 1 (*t* = 37) to July 31 (*t* = 157).

From Figure 8, we see that the epidemic peak is also delayed in this case. On the other hand, similar to the example for three months intervention, the epidemic size is effectively reduced only for *k* = 0.75.

**Figure 9.**
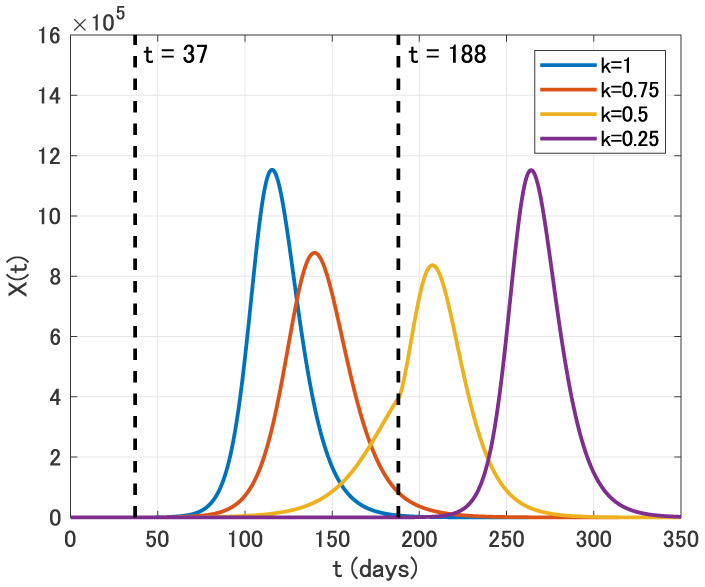
Time variation of the identification function *X*(*t*) for *k* = 1, 0.75, 0.5 and 0.25 in the five months intervention from April 1 (*t* = 37) to August 31 (*t* = 188).

We fourthly assume that the intervention is carried out for five months from April 1 (*t* = 37) to August 31 (*t* = 188). From Figure 9, we see that the epidemic peak is also delayed in this case. Moreover, the epidemic size is effectively reduced for *k* = 0.75 and *k* = 0.5. In contrast, the epidemic size for *k* = 0.25 is almost the same as that for *k* = 1.

**Figure 10.**
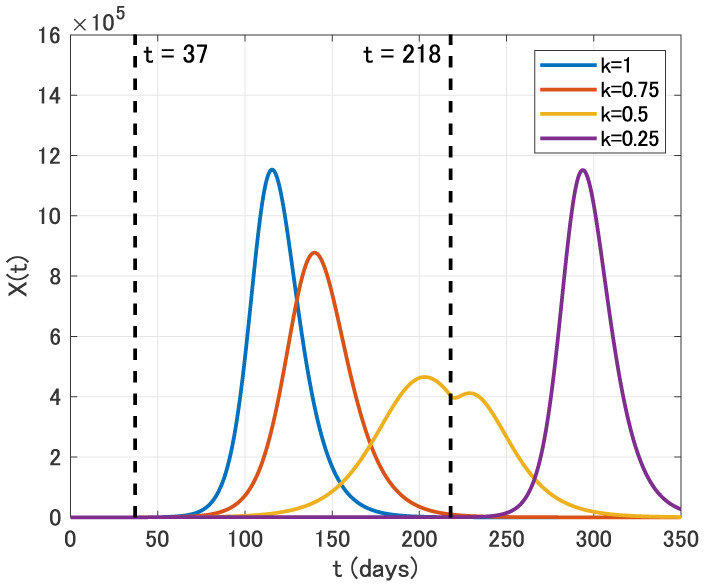
Time variation of the identification function *X*(*t*) for *k* = 1, 0.75, 0.5 and 0.25 in the six months intervention from April 1 (*t* = 37) to September 30 (*t* = 218).

We finally assume that the intervention is carried out for six months from April 1 (*t* = 37) to September 30 (*t* = 218). From Figure 10, we see that the epidemic peak is also delayed in this case. Moreover, the epidemic size is effectively reduced for *k* = 0.75 and *k* = 0.5. In contrast, the epidemic size for *k* = 0.25 is almost the same as that for *k* = 1.

From these examples, we obtain the following epidemiological insights.

- The epidemic peak is delayed monotonically as *k* decreases (that is, the contact rate is reduced by the intervention).
- The epidemic size is not necessarily reduced even if a long and strong intervention is carried out. To effectively reduce the epidemic size by the intervention, it suffices to continue the intervention until the epidemic peak attains during the intervention period.

**Figure 11.**
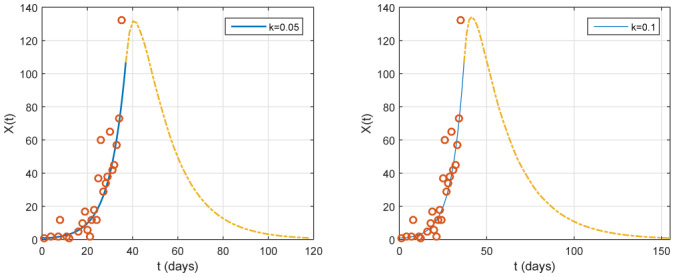
Case of a very strong intervention, with *ε* = 0.1. The red small circles are the reported case data. Left: time variation of the identification function *X*(*t*) for *k* = 0.05 in the three months intervention, from April 1 (*t* = 37). Right: time variation of the identification function *X*(*t*) for *k* = 0.1 in the four months intervention, from April 1 (*t* = 37).

**Figure 12.**
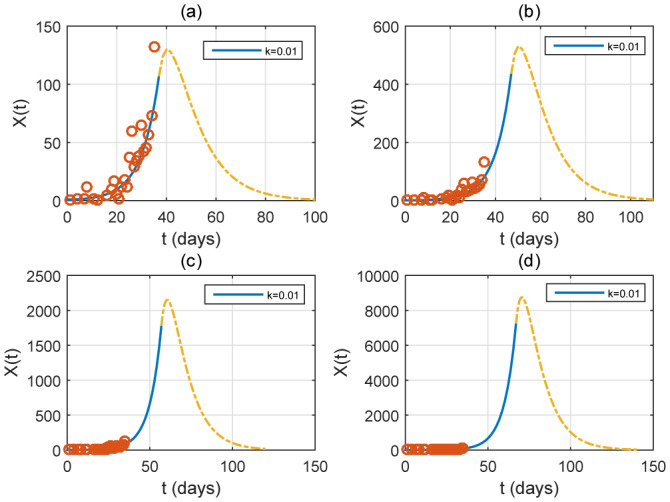
Simulation of different start times of carrying out the measures, with *ε* = 0.1 and *k* = 0.01 (Case of a very strong intervention). The red small circles are the reported case data. (a) from April 1 (*t* = 37), (b) from April 10 (*t* = 46), (c) from April 20 (*t* = 56) and (d) from April 30 (*t* = 66).

In Figure 11, we look at the case of measures that are more effective. Our model shows that the end of the disease can be reached in the three months intervention, from April 1, for *k* = 0.05 and in the four months intervention for *k* = 0.1, from April 1. More severe measures can induce an end of the disease in two months (see Figure 12 (a) with *k* = 0.01).

In Figure 12, we consider the cases where interventions are not taken early. It is shown that a delay in intervention implies a larger peak and additional duration for the epidemic to disappear. For instance, an intervention from April 20 implies a supplementary delay by 23 days for the epidemic to disappear with high considerable peak.

**Figure 13.**
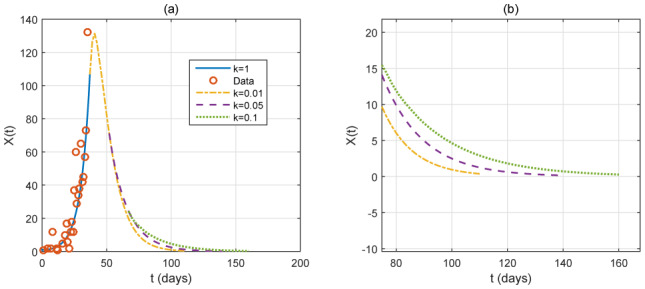
Simulation of the case where the strictness of intervention measures is reduced. The red small circles are the reported case data. From April 1 (*t* = 37), we take *k* = 0.01. From April 15 (*t* = 52), we take *k* = 0.05. From April 30 (*t* = 67), we take *k* = 0.1.

For some economical and social reasons and according to the situation of the epidemic, the strictness of intervention measures will decrease in a gradual way. The Figure 13 shows the case where the severity of intervention measures is reduced. This simulation suggests that decrease the parameter *k* gradually, on each half month, implies automatically a delay, at least of one month, for the end of the epidemic.

## 4 Conclusion

We have applied a mathematical model to predict the evolution of a COVID-19 epidemic in Algeria. It is employed to estimate the basic reproduction number *ℜ*_0_, to obtain the epidemic peak and to discuss the effect of interventions. In this model, we take the fact that the virus presents asymptomatic cases and that there exists a sufficiency lack of diagnostic test.

The prognostic capacity of our model requires a valid values for the parameters *β*, *λ*, *γ*, the mean incubation period 1/*λ* and the mean infectious period 1/*γ*. The precision of these parameters is very important for predicting the value of the basic reproduction number *ℜ*_0_ and the peak of the epidemic. Their estimations depend on the public health data in Algeria. To fight the new coronavirus COVID-19, it is necessary to control information based on valid diagnosis system.

From 25 February to 31 March, we founded that *ℜ*_0_ = 4.1 > 1, which means that we need strong interventions to reduce the epidemic damage that could be brought by the serious disease. Moreover, the model suggests that the pandemic COVID-19 in Algeria would not finish at a fast speed.

In the Figures 3, 4 and 5, where *ε* = 0.01, 0.05, 0.1, respectively, the data from the number of newly reported cases is well fitted the epidemic. The peak will occur at the month of June and approximately close to the middle, the maximum number of new cases (relatively also the cumulative number) could achieve an important value in Algeria. This number will probably persist at a high level for several days if we do not apply intervention measures (isolation, quarantine and public closings). The model's predictions highlight an importance for intervening in the fight against COVID-19 epidemics by early government action. To this end, we have discussed different intervention scenarios in relation to the duration and severity of these interventions. We see that the intervention has a positive effect on the time delay of the epidemic peak. On the other hand, the epidemic size is almost the same for short intervention (effective or not) and decrease depending on the severity of the measures. In contrast with the last previous case, we observe that a large epidemic can occur even if the intervention is long and sufficiently effective.

At the moment, the consequence of COVID-19 in China is encouraging for many countries where COVID-19 is starting to spread. Despite the difficulties, Algeria must also implement the strict measures as in Figure 11, which could be similar to the one that China has finally adopted.
